# Autochthonous leprosy in Spain: Has the transmission of *Mycobacterium leprae* stopped?

**DOI:** 10.1371/journal.pntd.0008611

**Published:** 2020-09-16

**Authors:** Inés Suárez-García, Diana Gómez-Barroso, Paul E. M. Fine

**Affiliations:** 1 Infectious Diseases Group, Department of Internal Medicine, Hospital Universitario Infanta Sofía, FIIB HUIS HHEN, Madrid, Spain; 2 Facultad de Ciencias Biomédicas y de la Salud, Universidad Europea, Madrid, Spain; 3 CIBERESP (CIBER en Epidemiología y Salud Pública), Centro Nacional de Epidemiología, Instituto de Salud Carlos III, Madrid, Spain; 4 Faculty of Epidemiology and Population Health, London School of Hygiene and Tropical Medicine, London, United Kingdom; Hospital Infantil de Mexico Federico Gomez, MEXICO

## Abstract

**Background:**

The aim of this study is to explore whether transmission of *M*. *leprae* has ceased in Spain, based upon the patterns and trends of notified cases.

**Methodology:**

Data on new cases reported to the National Leprosy Registry between the years 2003–2018 were extracted. In absence of detailed travel history, cases were considered “autochthonous” or “imported” based on whether they were born within or outside of Spain. These data were analyzed by age, sex, clinical type, country of origin, and location of residence at time of notification.

**Principal findings:**

Data were available on 61 autochthonous and 199 imported cases since 2003. There were clear declines in incidence in both groups, and more imported than autochthonous cases every year since 2006. Autochthonous cases were more frequently multibacillary and had older age at diagnosis compared to imported cases. All the autochthonous cases had been born before 1985 and were more than 25 years old at diagnosis. Male-to-female ratio increased with time for autochthonous cases (except for the last time period). The imported cases originated from 25 countries, half of them from Brasil and Paraguay. Autochthonous cases were mainly distributed in the traditionally endemic regions, especially Andalucía and the eastern Mediterranean coast.

**Conclusions:**

Autochthonous and imported cases have different epidemiologic patterns in Spain. There was a clear decline in incidence rates of autochthonous disease, and patterns consistent with those reported from other regions where transmission has ceased. Autochthonous transmission of *M*. *leprae* is likely to have now effectively stopped in Spain.

## Author summary

There is increasing interest in the potential for cessation of transmission of *M Leprae*. In several European countries, as social and economic conditions have improved, leprosy rates have declined and transmission appears to have stopped. The epidemiology of leprosy is still not fully understood: the study of leprosy in countries with low incidence rates can give valuable information on its epidemiology that could inform eradication campaigns in other countries. Leprosy has been endemic in Spain for centuries and, despite very low incidence in the last decades, new autochthonous cases are diagnosed every year. In this study, we analyse the epidemiologic trends of autochthonous leprosy in Spain in recent years. Our data provide insights into the dramatic decline of leprosy in Spain since 1950, and, for the cases diagnosed after 2003, show a shift from a predominace of disease attributable to locally acquired infection to overwhelming predominance of disease attributable to infections acquired elsewhere. The comparison of patterns of disease between the autochthonous and imported cases shows important differences, suggesting that the most recent autochthonous cases were those with long incubation periods after infections which were acquired many years ago. The epidemiologic trends are similar to those found in other countries with declining incidence and suggest that transmission effectively stopped in Spain more than two decades ago.

## Introduction

**Fig 1:**
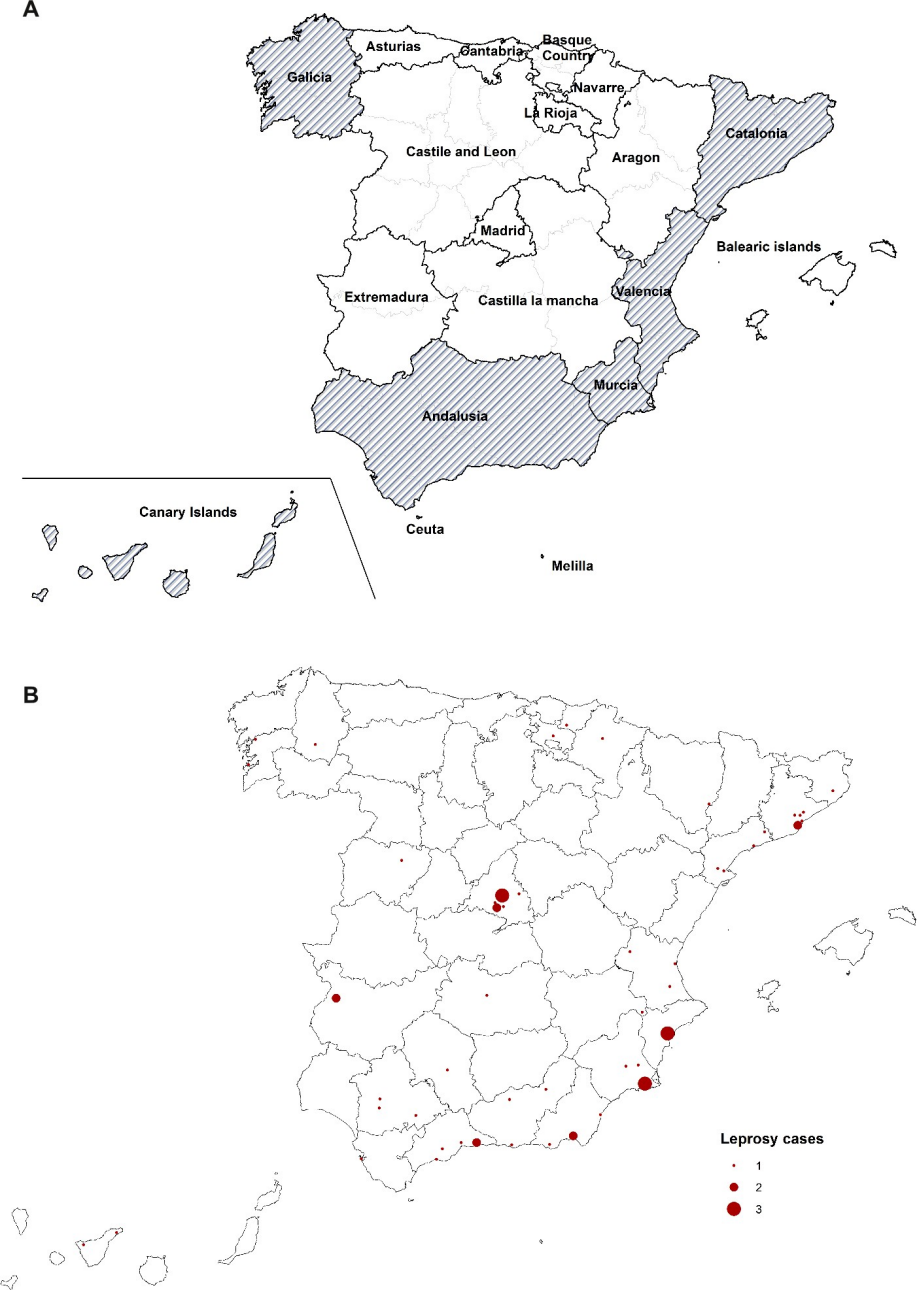
**A: Spanish autonomous regions and historical leprosy endemic regions B: Recorded location of residence of autochthonous cases, 2003–2018.** (A) Historical leprosy endemic regions are shown with diagonal shading. Division of provinces within each autonomous region is shown with light grey lines. (B) Village of residence (but not province of residence) was unknown for 7 cases resident in the provinces of Alicante (2 cases), Badajoz, Cádiz, Madrid, Murcia and Santa Cruz de Tenerife (1 case in each); these patients have been placed in the capital cities of each of their provinces in Fig 1B.

Leprosy has been endemic in Spain for centuries, with four historical areas of high prevalence: the eastern Mediterranean coast (which includes the regions of Cataluña, Valencia and Murcia), Andalucía in the south, Galicia in the northwest, and the Canary Islands (Fig 1A). Throughout the 19th century, the highest prevalence was found in the east coast, but this was surpassed by Andalucía during the second half of the 20th century[1,2]. It is unclear whether the higher prevalence in this region could be attributed to socioeconomic, environmental or other factors.

During the second half of the 20th century, incidence rates steadily declined in Spain, paralleling an increase in national gross domestic product[3]. This decline preceded the initiation of short-course multiple drug therapy, in the early 1980s[1]. The prevalence during the year 2018 was 0.0036 cases per 10,000 persons, well below the World Health Organization (WHO) prevalence target for the “elimination of leprosy as a public health problem”[4].

Studies in several countries with declining incidence rates of locally acquired leprosy have found consistent epidemiologic trends, such as changes in the geographic distribution (shifting to lower latitudes in Korea[5] and Japan[6]), increased proportions of cases with known family or household contacts, increased age at onset (associated with delayed infection and an increasing proportion of patients with long incubation periods), and increasing proportion of male cases and multibacillary (MB) forms[5–8].

During the last 15 years, most of the incident cases in Spain have been imported from countries with high leprosy prevalence[4,9,10]. However, apparent autochthonous cases (AC) are still being reported every year to the National Leprosy Registry[4,10], and several cases have been reported among Spanish-born patients with no history of travel to high prevalence countries[11–13], raising the question of whether transmission of *M*. *leprae* is still ongoing in the country. There have been few epidemiologic studies of leprosy in Spain in recent years, and most of them have focused on small regions without considering the geographic origin of the cases[14–16]. One recent study provided total numbers of autochthonous and imported cases, but focused on the incidence of imported cases by country of origin[9]. We have previously described autochthonous leprosy in the Valencia Region showing epidemiologic trends that suggest transmission has effectively stopped in this area[17], but whether this applies to the rest of Spain is unknown.

There is increased current interest in the potential for cessation of *M*. *leprae* transmission in different countries, stimulated by WHO and the Global Partnership for Zero Leprosy (GPZL)[18]. The European region is of particular interest in this regard, and WHO began reporting data from Europe in 2015[19]. The aim of this study was to describe the epidemiology of leprosy in Spain in recent years, with particular focus upon whether infection transmission still occurs in the country. Our hypothesis was that autochthonous transmission ceased in Spain in recent years.

## Methods

The numbers of leprosy cases reported in Spain since 1950 were obtained from the Annual Statistical Bulletin (*Anuario Estadistico de España*) of the National Institute of Statistics (*Instituto Nacional de Estadistica*, *INE*)[20]. For all leprosy cases notified between the years 2003 and 2018, the following additional data were obtained from the National Leprosy Registry: birth date, date of diagnosis, sex, village and province of residence, country of origin, and classification (multibacillary [MB]/paucibacillary [PB]). Cases were classified as “autochthonous” if their country of origin was Spain and “imported” if their country of origin was elsewhere. The National Leprosy Registry should include all patients diagnosed with leprosy in Spain, as leprosy notification is compulsory in the country. Although the National Leprosy Registry officially started in 1992, information on the patient’s country of origin, or other information identifying whether a case was attributable to local infection, was not available until 2003. Therefore, we pay most attention upon patients diagnosed with leprosy and notified to the registry from the years 2003 to 2018. The data were provided in an anonymized database in February, 2019.

Spanish population data, and numbers of migrant residents in Spain were extracted from the Spanish censuses provided by the National Statistics Institute[21]. For autochthonous cases, the village of residence was considered to be rural if the average population during the study period was <5,000 residents, and urban if it was ≥ 5,000 residents[22].

Descriptive analyses were carried out using frequency distributions or mean (standard deviation [SD]), as appropriate. Differences in proportions and means were assessed using χ^2^ test and Student’s t-test, respectively. All analyses were performed in Stata version 13.1 (StataCorp, College Station, TX, USA). Figures were produced with R version 3.2.5. Maps were produced with ARCGIS 10. The study was approved by the Ethics Committee of Hospital Universitario La Paz. Informed consent was not required as the data were extracted and analyzed anonymously.

## Results

**Fig 2:**
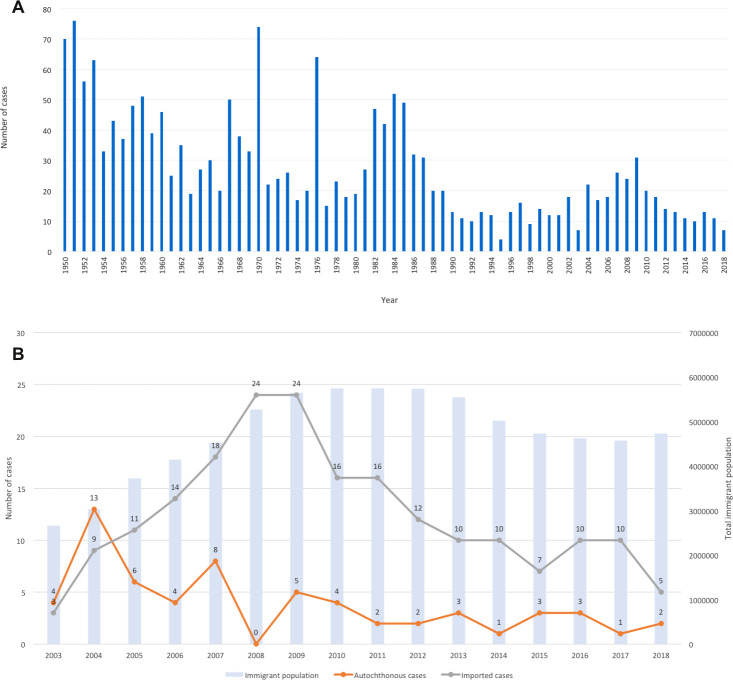
**A: Total numbers of leprosy cases notified in Spain by year since 1950. B: Total numbers of leprosy cases by place of birth (autochthonous vs imported) since 2003.** (B) Numbers of leprosy cases per year are shown by solid lines. Total immigrant population resident in Spain per year are shown by blue bars.

A total of 1900 leprosy cases were notified in Spain since 1950: the numbers of notified cases per year are shown in Fig 2A, showing a dramatic decline over the past 70 years. These numbers are broken down by place of birth (Spain or elsewhere) in Fig 2B for 260 patients notified since 2003: 61 born in Spain and 199 born elsewhere. Two patients notified after 2003 with no data on country of origin were excluded from the study.

The low numbers recorded for 2003 may be due to underreporting when the registration system was changed. Fig 2B also shows the total immigrant population resident in Spain, for every year since 2003. The number of imported cases per year increased from 2003–2008, simultaneous with a doubling of the total numbers of resident immigrants in the country. Of the cases born outside of Spain, 71 (36%) were from Brazil, 29 (15%) were from Paraguay, 15 (8%) and 14 (7%) were from Bolivia and Colombia respectively, with smaller case numbers from 21 other countries. Numbers of notified cases considered autochthonous declined dramatically from 2003, and the same was seen among imported cases from 2008.

Table 1 shows the annual incidence rates of autochthonous and imported cases during the four time periods analyzed. Table 2 shows the characteristics of autochthonous compared to imported cases. In both groups, patients were predominantly male, and MB. Compared with imported cases, autochthonous cases had an older age at diagnosis. Also, a higher proportion of MB patients was seen among autochthnous cases (79% of the autochthonous cases were MB compared to 62% of the imported cases; p = 0.017).

**Table 1:** Number of leprosy cases, annual incidence rates per 100,000 person-years, and average annual population for each time period.

Time period	Autochthonous cases			Imported cases		
	N	Incidence (95% CI)	Population	N	Incidence (95% CI)	Population
2003–06	27	0.017 (0.011–0.024)	40,289,743	37	0.273 (0.192–0.376)	3,393,317
2007–10	17	0.010 (0.006–0.017)	40,985,169	82	0.387 (0.308–0.480)	5,296,180
2011–14	8	0.005 (0.002–0.009)	41,574,867	48	0.218 (0.160–0.289)	5,514,367
2015–18	9	0.005 (0.002–0.010)	41,955,195	32	0.172 (0.117–0.242)	4,663,930
Total	61			199		

95% CI: 95% confidence interval.

**Table 2:** Proportion of MB cases and male-to-female ratio for autochthonous and imported cases in Spain, 2003–2018.

Time period	Autochthonous cases			Imported cases		
	N	MB/total (%)	M/F ratio	N	MB/total (%)	M/F ratio
2003–06	27	23/27 (85%)	17/10 = 1.7	37	21/32* (66%)	25/12 = 2.1
2007–10	17	15/17 (88%)	12/5 = 2.4	82	48/78* (62%)	46/36 = 1.3
2011–14	8	5/6* (83%)	7/1 = 7.0	48	27/46* (59%)	17/31 = 0.5
2015–18	9	3/8* (38%)	3/6 = 0.5	32	20/30* (67%)	17/15 = 1.1
Total	61	46/58* (79%)	39/22 = 1.8	199	116/186* (62%)	105/94 = 1.1

*Classification not available for several cases

MB: multibacillary cases. M/F ratio: male-to-female ratio.

**Fig 3:**
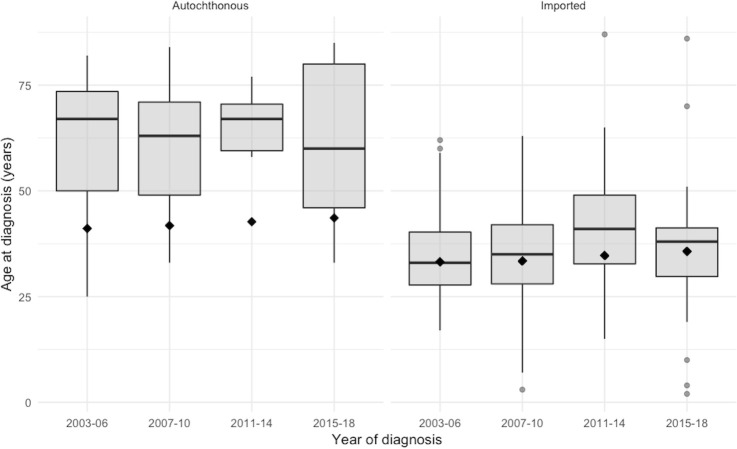
Age at diagnosis by year of diagnosis in autochthonous and imported cases during the four time periods analyzed. Diamonds are the average age of the native-born and immigrant population for each time period.

Fig 3 shows the frequency distributions of autochthonous and imported cases by age over the years 2003–2018. The autochthonous cases were appreciably older: their average age was 65 (SD: 15) years compared with 36 (SD:13) years for imported cases (p < 0.001), with little evidence of age shifts over the period examined. Seven of the most recently born autochthonous cases were born in the 1970s and only one after 1980 (a female who was born in 1985 and diagnosed with leprosy in Cataluña aged 33 years in 2018). The youngest was a 25 years old male born in 1978. For imported patients, the latest birth date was 2016 and the youngest patient was diagnosed at 2 years of age.

Patients with autochthonous leprosy were predominantly male (64% of the cases), and the male-to-female ratio increased with time for the first three time periods analysed and decreased in the last period, but the number of patients in the last two periods was low (Table 2). There was no clear trend seen on the male-to-female ratio among patients with imported leprosy.

The proportion of MB patients in successive 4-year time periods is shown in Table 2. Among the 244 patients with information on MB/PB classification, 79% of the autochthonous and 62% of the imported cases had MB leprosy. The average age at diagnosis was 62 (SD 15) years for cases with MB leprosy and 58 (SD 16) years for cases with PB leprosy. For autochthonous cases, the proportion of cases with MB did not differ significantly between males and females: 81.1% of the males and 79.3% of the females had MB leprosy(p = 0.659). However, for imported cases the proportion of cases with MB leprosy was significantly higher among males: 69.4% of the males and 54.5% of the females had MB leprosy (p = 0.037).

The geographic distribution of autochthonous cases is shown in Fig 1B. Cases were found mainly in the traditional endemic areas: the Mediterranean coastline (including Cataluña, Valencia, and Murcia) and Andalucía, with a few isolated cases in other traditionally endemic areas (Galicia and the Canary Islands), as shown in the Figure. In addition, several cases were diagnosed in the Madrid metropolitan area. There were few cases reported from other regions. Ten (16.4%) of the autochthonous cases were resident in rural areas at date of notification.

## Discussion

These data provide insights into the dramatic decline of leprosy in Spain since 1950. Similar to the pattern seen elsewhere in Europe and in many other high income countries, there was a shift from a predominance of disease attributable to locally acquired infection to overwhelming predominance of disease attributable to infections acquired elsewhere[23,24]. The comparison of patterns of disease between these two groups, identified on the basis of country of birth, is revealing.

We appreciate several limitations to the data. First, there was no information on travel history for most patients, and therefore we cannot exclude that some of the cases born in Spain might have acquired the infection elsewhere and that some of the foreign-born cases could have acquired their infections in Spain. We note that this issue arises often in studies of leprosy, and is related to the fact that WHO has encouraged countries to report cases by whether born in the reporting country or elsewhere, since 2015[19]. Second, due to the information available in the national registry, our time period was restricted to the years 2003–2018, a short time span for analysis of temporal trends. Third, we had no information on known leprosy contacts for most patients and therefore we could not analyse the role of family or community contacts in the epidemiology of the disease. Fourth, we note that the criteria for classifying some cases as MB may have been based upon traditional criteria involving histopathology, and not the current clinical criterion of more than 5 lesions. Also, there was no information on the MB/PB classification for 6% of the patients. Finally, a limitation of all studies done with leprosy registries is the possibility of underestimation of new cases due to underdiagnosis (because health workers may not be familiar with the disease as it becomes less prevalent) or underreporting (though this is unlikely as notification of new leprosy cases is compulsory in Spain). We noted a low number of cases reported in 2003, which might have been due to a change in the reporting system in that year. However, the trends were consistent in the following years and we do not think this has greatly affected our results.

Depite these caveats, the extraordinary decline in recent decades is convincing and consistent with patterns which occurred in northern Europe decades ago and more recently in high income countries such as Japan and Korea, as well as in northern Portugal[5,6,25]. The increasing predominance of imported cases and its correlation with the numbers of immigrants living in the country is as expected[9]. The increase in imported leprosy incidence beween 2003 and 2008 was driven largely by Brazilian and Bolivian cases. Brazil has a high leprosy prevalence (second highest in the world) and the numbers of Brazilian and Bolivian residents in Spain increased by 165% and 195%, respectively, between the time periods 2003–6 and 2007–10, compared to an increase of 61% of all the migrant populations residing in Spain over these years [21]. The fact that leprosy incidence among migrants has been found to be the highest during the first years upon arrival to their country of destination and falls sharply thereafter[26,27] is consistent with the likelihood that these cases were attributable to infections contracted in their countries of origin. We could not determine the interval from arrival to diagnosis in our imported cases, as there is no information on date of arrival in the National Leprosy Registry.

The cases among individuals born in Spain are most relevant for assessing whether transmission has occurred in recent times in the country. The autochthonous cases had significantly older age at diagnosis than the imported ones, and their average age was appreciably older than the average age of the Spanish population for the same time period. Although the time span of our study was short, age at onset increased over successive time periods (except for the last period, when the number of cases was small), and this trend was not found among imported cases. The average age at diagnosis for autochthonous cases was 65 years old, and there were no autochthonous cases younger than 25 years old. This pattern of increasing age at diagnosis has been described consistently in other regions with declining incidence rates[5–8,28,29], suggesting that the most recent cases are those with long incubation periods after infections which were acquired many years ago[7]. The median estimated incubation period of leprosy is 2–5 years for PB forms and 8–12 years for MB forms, but the incubation period can be longer than 20 years in some patients [30]. The high proportion of MB cases, which have longer incubation periods, is consistent with the increasing age at diagnosis and has been described in other studies from regions with declining incidence rates[7]. On this basis, given the small and declining numbers of cases diagnosed since 2003, and the fact that there were no autochthonous cases born after 1985, one might argue that autochthonous transmission stopped around 1990.

The male-to-female ratio increased with time among autochthonous cases (except for the last period, which had a low number of cases), whereas it showed no clear trend among imported cases. An increasing male-to-female ratio has been described in other populations with declining leprosy incidence, and has been associated with an increasing proportion of MB forms (which are more frequent among males)[7,8,31].

The geographic location of the autochthonous cases largely reflects the regions where leprosy was traditionally endemic in Spain, predominantly the eastern Mediterranean coast and Andalucía. Several cases were diagnosed in the city of Madrid or its surrounding areas, which was not a traditionally endemic region, and we suspect these are attributable to migration to the capital city from other regions. Other studies have shown a shift towards lower latitudes as incidence declines[5,6,8], and we have previously published a preference for coastal areas in a study in the Valencia Region[17], which has also been shown in Portugal[28] and Norway[29]. We did not find any consistent changes in the geographic distribution of autochthonous cases over the short period of this study.

Although our study in the Valencia Region[17] and others[29,30,32] have suggested a predilection for rural rather than urban areas, we did not find a similar pattern in this study. The proportion of cases residing in rural areas was 16.4%, compared with 16.2% of the general Spanish population in the study period[33]. However, our results on the geographic distribution and the rural/urban setting are limited because the only information available in the registry was on the place of residence, and not on the place of birth. It may be that some patients acquired the infection as children in rural areas, but then migrated to urban areas, a movement pattern which was common in Spain during the second half of the 20^th^ century. This could also explain the cluster of cases diagnosed in the area of Madrid.

The differences between cases born within and outside Spain are consistent with the hypothesis that they were infected locally versus elsewhere, respectively, and with transmission having effectively stopped in Spain more than two decades ago. The possibility of transmission from imported cases within Spain is not zero, but experience has shown it to be rare in high income countries (the last reported transmission from an imported case in the UK was in 1954[34]). The explanation for this decline must include the impact of control measures–case finding and treatment and BCG vaccination–but it has doubtless been assisted greatly by general impovement in socio-economic conditions; changes in environmental conditions might have also played a role. The biological mechanisms have not yet been elucidated: whether the decline of leprosy incidence is attributable to a lower risk of acquiring the infection with *M*. *leprae*, or the failure of new infections to progress to clinical disease, is not entirely clear. Without a valid test for (subclinical) *M*. *leprae* infection this crucial question of leprosy’s natural history will remain unanswered.
